# Facile Synthesis Bi_2_Te_3_ Based Nanocomposites: Strategies for Enhancing Charge Carrier Separation to Improve Photocatalytic Activity

**DOI:** 10.3390/nano11123390

**Published:** 2021-12-14

**Authors:** Di Wu, Jun Guo, Zhen-Hua Ge, Jing Feng

**Affiliations:** Faculty of Materials Science and Engineering, Kunming University of Science and Technology, Kunming 650093, China; 20202230034@stu.kust.edu.cn (D.W.); 20183130004@stu.kust.edu.cn (J.G.); jingfeng@kust.edu.cn (J.F.)

**Keywords:** core-shell, heterostructure, photocatalysis, Bi_2_Te_3_ composites

## Abstract

Varying structure Bi_2_Te_3_-based nanocomposite powders including pure Bi_2_Te_3_, Bi_2_Te_3_/Bi core−shell, and Bi_2_Te_3_/AgBiTe_2_ heterostructure were synthesized by hydrothermal synthesis using Bi_2_S_3_ as the template and hydrazine as the reductant. Successful realization of Bi_2_Te_3_-based nanostructures were concluded from XRD, FESEM, and TEM. In this work, the improvement in the performance of the rhodamine B (RhB) decomposition efficiency under visible light was discussed. The Bi_2_Te_3_/AgBiTe_2_ heterostructures revealed propitious photocatalytic performance ca. 90% after 60 min. The performance was over Bi_2_Te_3_/Bi core-shell nanostructures (ca. 40%) and more, exceeding pure Bi_2_Te_3_ (ca. 5%). The reason could be scrutinized in terms of the heterojunction structure, improving the interfacial contact between Bi_2_Te_3_ and AgBiTe_2_ and enabling retardation in the recombination rate of the photogenerated charge carriers. A credible mechanism of the charge transfer process in the Bi_2_Te_3_/AgBiTe_2_ heterostructures for the decomposition of an aqueous solution of RhB was also explicated. In addition, this work also investigated the stability and recyclability of a Bi_2_Te_3_/AgBiTe_2_ heterojunction nanostructure photocatalyst. In addition, this paper anticipates that the results possess broad potential in the photocatalysis field for the design of a visible light functional and reusable heterojunction nanostructure photocatalyst.

## 1. Introduction

Environmental and energy issues have always been the focus of our attention. However, energy is consumed in large quantities and has led to a series of environmental problems in recent decades [1,2]. Therefore, it is vital to develop efficient catalysts for the degradation of organic pollutants and water splitting or the reduction in CO_2_. Semiconductor-based photocatalytic materials have been extensively investigated as a hot research topic because of their unique chemical and physical properties and potential applications [3,4,5]. Their excellent properties and potential applications have something to do with the morphologies, dimensions, and structures of the nanomaterials.

First, the decolorization efficiency of organic pollutants was carried out to evaluate the performance of photocatalysts. There are many photochemical reactions involved in the whole degradation process. During the photocatalytic process, it must be noted that two main reactions are bound to occur for the successful production of reactive oxidizing species to yield: one is the oxidation of dissociatively adsorbed H_2_O by photogenerated holes, and the other is the reduction in an electron acceptor (such as dissolved molecular oxygen) by photoexcited electrons. Before the two reactions occur, we need sufficient photogenerated electrons and hole pairs to exist; however, the recombination of photogenerated charge carriers is still the major limitation in semiconductor photocatalysis [6,7,8]. Herein, the design of one-dimensional nanostructures (such as nanorods, nanotubes, nanowires, and nanoribbons, etc.) has been shown to possess inherent merits including higher specific surface area and fast collection of photoinduced charge carriers [9,10,11,12]. Xueqin Liu et al. focused on the preparation and properties that significantly advanced noble metal (Au, Ag, Pt, and Pd)-metal oxide nanohybrids including noble metal-decorated metal oxide nanoparticles and nanoarrays, core/shell and yolk/shell nanostructures such as nanoplates, nanowires, nanotubes, etc. [13]. Indeed, the noble metal cooperates with the special structure to enhance the solar energy conversion, and the performance could also achieve a satisfactory result with increasing noble metal content. However, the appropriate photocatalytic performance sacrifices many noble metals, is uneconomical, and is not environmentally friendly. Leiming Lang et al. investigated the decomposition efficiency of TiO_2_ 1D nanostructures: solid, hollow, tube-in-tube fibers. Certainly, the photodegradation ratio is superior to commercially available powders. Nevertheless, the absorption of light by these nanofibers is confined to the UV region. Although it still possesses better photodegradation activity under visible light irradiation, it is not as good as decomposition under UV light, which limits the application of bare TiO_2_. Therefore, for the synthesis of nanostructured materials, semiconductor heterostructures and core-shell nanoparticles reveal another funny and attractive direction [14,15]. Bismuth telluride has been utilized for the conversion between heat and electricity for decades and has strong absorption of visible light, low toxicity, stability, and economic value. Furthermore, a promising photocatalyst could be obtained by designing different structures to improve the separation of photoinduced charge carriers.

Bi_2_Te_3_ is an intrinsically layered structure and shows flake-like morphology in the common growth conditions. Pure Bi_2_Te_3_ has no photocatalytic properties, and Bi_2_Te_3_ with a rod-like morphology was obtained by ion-exchange using Bi_2_S_3_ as a template during the hydrothermal process [16,17]. Bi_2_Te_3_-based catalysts with different microstructures were obtained by microstructure control. It is known that the microstructure plays an important role in photocatalytic decomposition [18,19]. The Bi_2_Te_3_/AgBiTe_2_ heterostructure powders synthesized in this work showed an excellent photocatalytic property. The photocatalytic mechanism of heterostructure powders are discussed in detail.

## 2. Experimental

### 2.1. Synthesis

Chemical reagents directly used in this study were analytically pure. All powders were synthesized by two hydrothermal method steps as described in previous works [20,21,22]. Pure Bi_2_Te_3_ and Bi_2_Te_3_/Bi core-shell NRs were synthesized by Bi_2_S_3_ NRs and tellurium powders with molar ratios of 1:3 and 1:2.5 under a hydrothermal process. In addition, by keeping the raw materials the same, with a molar ratio of 1:2.5 and 5% molar or 10% molar AgNO_3_ powder added under a hydrothermal process, the Bi_2_Te_3_/AgBiTe_2_ heterostructure could be obtained. The hydrothermal reaction conditions were 180 °C for 6 h to obtain NRs with different structures.

### 2.2. Characterization

The powders were checked by X-ray diffraction (XRD) with Cu-Kα radiation. The morphologies of Bi_2_Te_3_-based powder samples were analyzed by field emission scanning electron microscopy (FESEM); in addition, the morphologies and microstructure were further examined using transmission electron microscopy (TEM). The UV–Vis absorption spectra of the samples were characterized to obtain the band gap energy (*E_g_*) by a UV–Vis–NIR spectrophotometer (UV-3600 plus). The determination of *E_g_* by applying the Kubelka–Munk (*K*−*M*) method can reach a great advantage [23]. The *K–M* method is based on the following equation: *F*(*R*) = (1 − *R*)/2*R, *R* is the reflectance, and *F*(*R*) is proportional to the extinction coefficient (*α*) and the absorbance (*A*). The photocatalytic reactions were evaluated by the degradation of rhodamine B (RhB) from Guangzhou Howei Chemical Co. Ltd. under visible light irradiation with a 500 W xenon lamp. The photocatalytic oxidation decomposition of RhB by Bi_2_Te_3_-based composite powders was examined with a UV–Vis–NIR spectrophotometer.

### 2.3. Techniques of Catalysis

In this experiment, Bi_2_Te_3_-based nanopowders as catalysts (fixed weight of 0.05 g) were added to a mixed solution of organic pollutants (200 mL, 2.5 mg/L). The circulating water of the cylindrical quartz vessel must keep flow in the whole reaction process, which can take the quantity of heat from the lamp to ensure the reaction at room temperature and decrease the thermal catalytic effect as much as possible. Moreover, the mixture was placed in a special black box and stirred for 1 h. On one hand, the type of irradiation light was chosen to avoid the unnecessary light effect, and on the other hand, the adsorption–desorption equilibrium between the organic pollutant and photocatalyst was reached before light exposure. The dye degradation was monitored from the intensity of the absorption peak of RhB relative to its initial intensity by measuring the UV–Vis absorbance at a certain time interval after irradiation on the basis of the formula *η* = (*A*_0_ – *A*)/*A*_0_ × 100%, where *η* is the decolorization efficiency of the reaction; *A_0_* is the initial absorbance of the RhB solution before the light; and *A* is the absorbance of the RhB solution at a given time.

## 3. Results and Discussion

**Figure 1:**
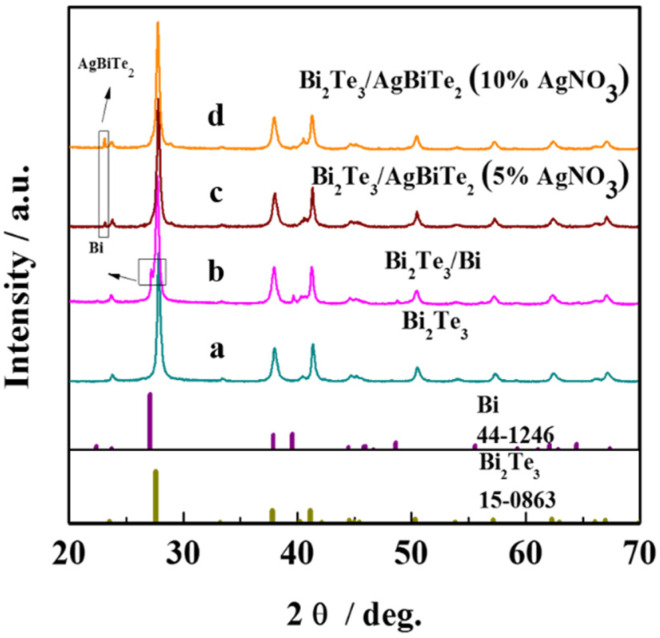
XRD patterns of the products prepared at 180 °C for 6 h (**a**) pure Bi_2_Te_3_, (**b**) Bi_2_Te_3_/Bi core-shell, (**c**) Bi_2_Te_3_/AgBiTe_2_ heterostructure (5% AgNO_3_), and (**d**) Bi_2_Te_3_/AgBiTe_2_ heterostructure (10% AgNO_3_).

The X-ray diffraction (XRD) patterns of Bi_2_Te_3_-based samples synthesized by the hydrothermal method at 180 °C for 6 h are shown in Figure 1. As the hydrothermal reaction was completed, the diffraction peaks of the main phase were well matched with Bi_2_Te_3_ (PDF#15–0863), except for the two impure phases that appeared with a molar ratio of 1:2.5, which were verified as Bi (PDF#44–1246) and AgBiTe_2_ (PDF#18–1172). The XRD pattern can be indexed to pure Bi_2_Te_3_ with a starting Bi_2_S_3_-to-Te ratio of 1:3 (shown at the bottom XRD line in Figure 1). With increasing AgNO_3_ content in the starting material, the AgBiTe_2_ XRD lines became more prominent, suggesting an increasing amount of AgBiTe_2_ with a decreasing amount of Bi in the final product, as shown in Figure 1b–d. Therefore, Bi_2_Te_3_/Bi core-shell NRs can be obtained at a Bi_2_S_3_-to-Te ratio of 1:2.5 without AgNO_3_ powder added in the whole hydrothermal process. In addition, a Bi_2_Te_3_/AgBiTe_2_ heterostructure was also produced as the Bi shell disappeared [24].

There is a possible reaction process for the formation of Bi_2_Te_3_, and Bi_2_Te_3_/Bi and Bi_2_Te_3_/AgBiTe_2_ NRs can be rationally interpreted as follows (1)–(7):

(1) $$\mathrm{Te}+{\mathrm{NH}}_{2}{\mathrm{NH}}_{2}+{\mathrm{OH}}^{-}\to {\left({\mathrm{Te}}_{3+1}\right)}^{2-}+N_{2}+H_{2}O$$

(2) $${\left({\mathrm{Te}}_{3+1}\right)}^{2-}\to 3\mathrm{Te}+{\mathrm{Te}}^{2-}$$

(3) $${\mathrm{Bi}}_{2}S_{3}+{\mathrm{NH}}_{2}{\mathrm{NH}}_{2}\to \mathrm{Bi}+N_{2}+H_{2}S$$

(4) $${\mathrm{Bi}}_{2}S_{3}+3{\mathrm{Te}}^{2-}\to {\mathrm{Bi}}_{2}{\mathrm{Te}}_{3}+3S^{2-}$$

(5) $$2\mathrm{Bi}+3\mathrm{Te}\to {\mathrm{Bi}}_{2}{\mathrm{Te}}_{3}$$

(6) $${\mathrm{Bi}}_{2}{\mathrm{Te}}_{3}+{\mathrm{NH}}_{2}{\mathrm{NH}}_{2}\to \mathrm{Bi}+N_{2}+H_{2}\mathrm{Te}$$

(7) $${\mathrm{Bi}}_{2}{\mathrm{Te}}_{3}+\mathrm{Bi}+{\mathrm{Ag}}^{+}\to {\mathrm{AgBiTe}}_{2}$$

First, highly reactive Te and Te^2−^ ions formed by the original Te powders react with NaOH and hydrazine, as shown in Equations (1) and (2) [25]. This reactive Te differs from the raw Te powder used as the Te source, which can react with Bi directly. Next, in Equation (3), bismuth ions (Bi^3+^) in Bi_2_S_3_ were reduced to metallic Bi by hydrazine, and Te was more prone to being reduced to Te^2−^ by hydrazine under alkaline conditions [26]. There are two steps for the formation of Bi_2_Te_3_: one is ion exchange between Te^2−^ and S^2−^ in the mixed solution, as shown in Equation (4), and the other is the direct reaction of Bi and Te with enough Te (Bi_2_S_3_-to-Te ratio of 1:3 in Equation (5)). However, as in the reduction case for Bi_2_S_3_, Bi_2_Te_3_ can also be reduced to Bi by hydrazine (Equation (6)). Obviously, there is a theoretical balance between the formation and dissolution of Bi_2_Te_3_ on the surface of the NR. However, as the ratio became 1:2.5, which means that the amount of Te was not enough to form Bi_2_Te_3_, a Bi_2_Te_3_/Bi core-shell structure was produced. Moreover, when AgNO_3_ was added, released silver ions reacted with the superficial Bi shell and Bi_2_Te_3_ core of the NRs to obtain Bi_2_Te_3_/AgBiTe_2_ heterojunction coupling.

**Figure 2:**
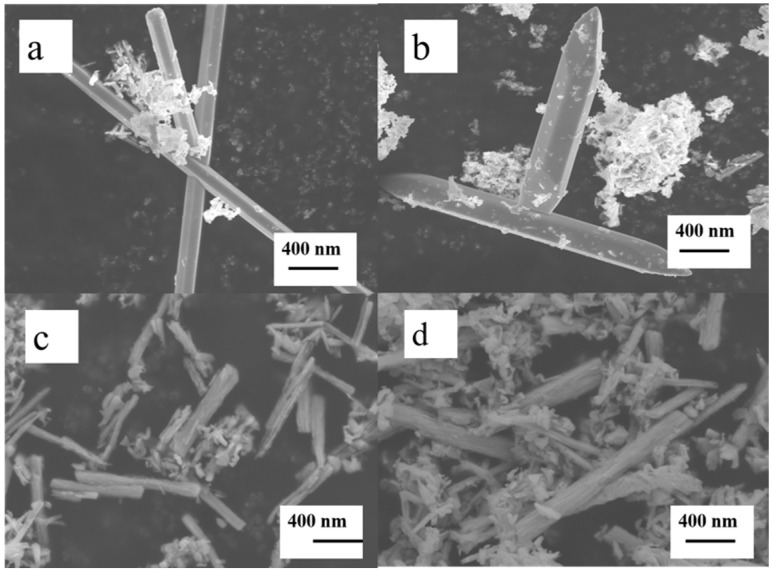
FESEM images of (**a**) pure Bi_2_Te_3_, (**b**) Bi_2_Te_3_/Bi core-shell, and (**c**,**d**) Bi_2_Te_3_/AgBiTe_2_ heterostructure.

To gain insight into the morphology and surface features of the resultant samples, we utilized FESEM studies. As shown in Figure 2a, the morphology of pure Bi_2_Te_3_ was a smooth surface rod-like with an average particle size of 100–2200 nm in diameter. In addition, as the Te powders were limited, the surface layer of pure Bi_2_Te_3_ was reduced to Bi by hydrazine, and then Bi, similar to a cloth, encapsulated the core Bi_2_Te_3_. The FESEM image of the Bi_2_Te_3_/Bi core-shell also showed essentially the same smooth surface morphology composed of rod-type particles. Furthermore, varying percentages of AgNO_3_ were added to synthesize the Bi_2_Te_3_/Bi core-shell, and then AgBiTe_2_ yielded the surface of Bi_2_Te_3_ by chemical reaction. Herein, the surface became rough because plenty of salient (the salient means the generated AgBiTe_2_, see TEM for details) grew on it, as revealed in Figure 2c,d.

**Figure 3:**
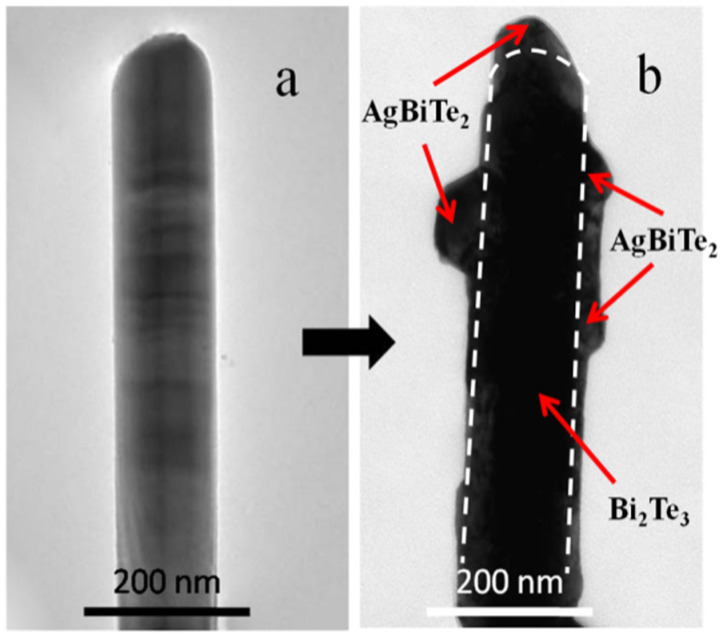
TEM images of (**a**) pure Bi_2_Te_3_ and (**b**) Bi_2_Te_3_/AgBiTe_2_ heterojunction nanostructures.

In more detail, the enlarged TEM image in Figure 3 shows that the pure Bi_2_Te_3_ (a) and Bi_2_Te_3_/AgBiTe_2_ heterojunction NRs (b) were approximately 130 nm in size. In addition, pure Bi_2_Te_3_ was rod-like with a smooth surface; however, the TEM results portrayed in Figure 3b illustrate the AgBiTe_2_ junction of an average size of 40 nm scattered onto the surface of a Bi_2_Te_3_ NR, pointing toward evidence of heterogeometry in the Bi_2_Te_3_/AgBiTe_2_ nanostructures.

**Figure 4:**
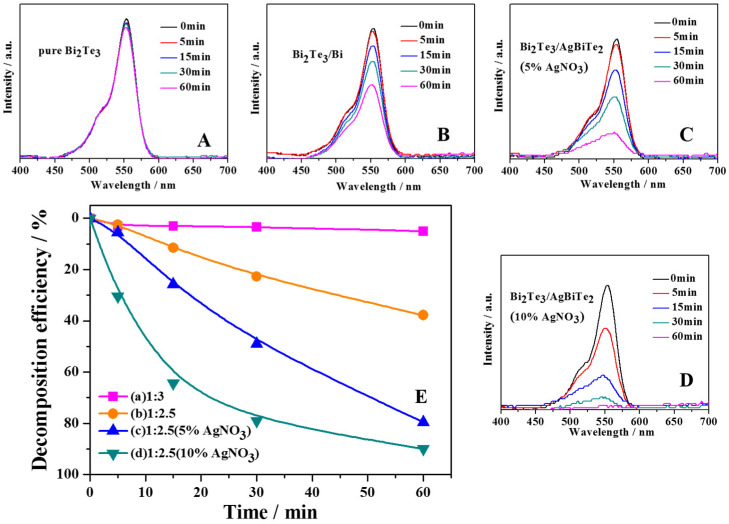
Uv–Vis absorption spectra of RhB solution with (**A**) pure Bi_2_Te_3_, (**B**) Bi_2_Te_3_/Bi NR, (**C**) Bi_2_Te_3_/AgBiTe_2_ NR (with 5% AgNO_3_), (**D**) Bi_2_Te_3_/AgBiTe_2_ NR (with 10% AgNO_3_), and the relationship between the structure of the powder and the degradation rate of RhB (**E**).

The Beer–Lambert law suggests that the concentration of RhB solution and the absorption intensity of the UV–Vis absorption peak are closely related. *A = abc*, where *a* is the absorption coefficient of the solution; *b* is the thickness of the colorimetric ware; and *c* is the concentration of the RhB solution at a certain time. The absorbance of the solution during the photocatalysis process can be utilized as a sign to characterize the efficiency of the photocatalyst for the decomposition of the dye. Figure 4 shows the absorption spectra of RhB solutions photocatalyzed by Bi_2_Te_3_ nanopowders (Figure 4A–D) along with the degradation rate curves of RhB (Figure 4E). First, pure Bi_2_Te_3_ NRs were prepared with a starting Bi_2_S_3_-to-Te ratio of 1:3 as the photocatalyst, and negligible decomposition was seen in Figure 4A, proving that pure Bi_2_Te_3_ could hardly degrade the RhB solution under light irradiation. For Bi_2_Te_3_/Bi core-shell NRs prepared with a Bi_2_S_3_-to-Te ratio of 1:2.5, less than 40% of the RhB solution was degraded in 60 min. In addition, the Bi_2_Te_3_/AgBiTe_2_ heterostructure NRs prepared with a Bi_2_S_3_-to-Te ratio of 1:2.5 and 5% AgNO_3_ were added, and the degradation rate reached ca. 80% after 60 min illumination. In particular, the Bi_2_Te_3_/AgBiTe_2_ heterostructure NRs prepared from a Bi_2_S_3_-to-Te ratio of 1:2.5 with 10% AgNO_3_ exhibited significantly higher photocatalytic efficiency, and the degradation rate reached ca. 80% in 30 min, and the absorption curve became a straight line after 60 min, indicating that more than 90% of the RhB solution degraded after a reaction time of 60 min.

**Figure 5:**
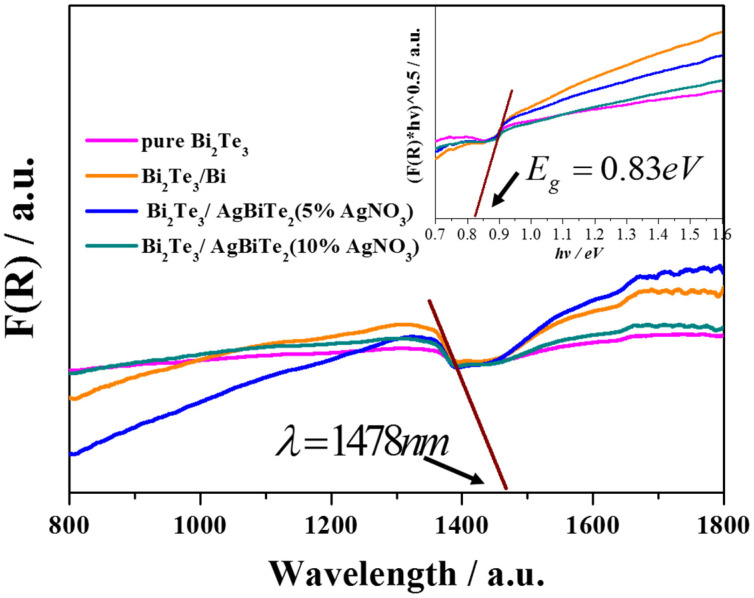
UV–Vis absorption spectra and diffuse-reflectance UV–Vis spectrum (the right upper inset) of Bi_2_Te_3_ powders.

The degradation rate curves in Figure 4E were calculated from the absorption intensities from Figure 4A–D, which corresponded to pure Bi_2_Te_3_ (a), Bi_2_Te_3_/Bi core-shell NRs (b), Bi_2_Te_3_/AgBiTe_2_ heterostructure NRs with 5% AgNO_3_ (c), and NRs with 10% AgNO_3_ (d). Obviously, the RhB solution of pure Bi_2_Te_3_ degraded showed little change under visible light irradiation after 60 min. Surprisingly, as shown in Figure 4E, compared to pure Bi_2_Te_3_ photocatalyst powders, samples b, c, and d exhibited amazing photocatalytic activity, especially samples b and d, which could degrade at least 80% of the initial RhB dye under the same conditions. Compared to sample c, clearly in d, the decomposition efficient value enhanced quickly to approximately 40% after 30 min. In an effort to observe the band gap variation, and the light capture ability of these samples, the UV–Vis absorption spectra and diffuse reflectance UV–Vis spectrophotometer of the samples are shown in Figure 5. Significantly, not only the absorption edge but also the band gap did not shift and were found to be ca. 1478 nm and 0.83 eV, respectively, which indicated that the photo absorption area and the photon utilization efficiency had hardly changed. These results show that the structure of Bi_2_Te_3_ powders plays an important role in photocatalytic activity in RhB solution.

**Figure 6:**
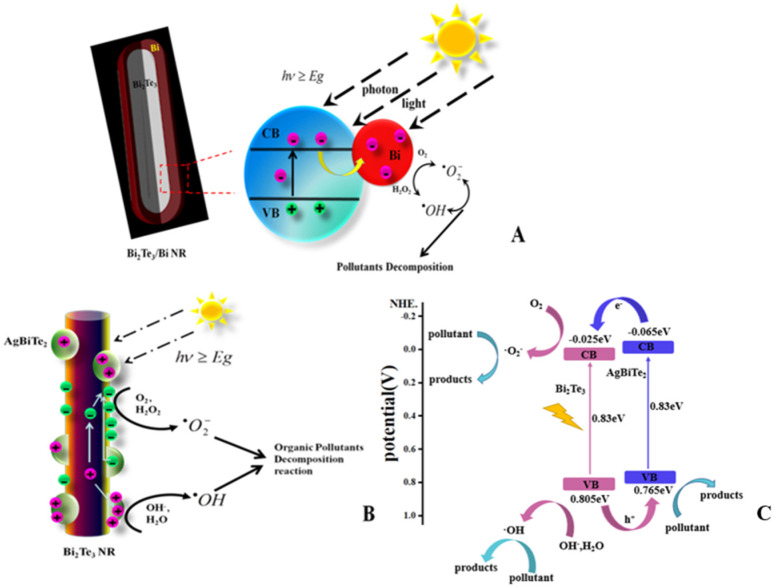
Schematic illustration of the mechanism for the photocatalysis by (**A**) Bi_2_Te_3_/Bi core−shell NRs. (**B**) Bi_2_Te_3_/AgBiTe_2_ heterostructure NRs materials under vis-light irradiation. (**C**) Schematic illustration of the mechanism for the photocatalysis by the Bi_2_Te_3_/AgBiTe_2_ heterostructure under visible irradiation.

A variety of experiments revealed the photocatalytic decomposition of RhB solutions with Bi_2_Te_3_ NRs with various structures. Speculatively, the possible major reaction steps involved in the photocatalytic mechanism of Bi_2_Te_3_ semiconductors and Bi_2_Te_3_-based composites are summarized as follows (see Equations (8)–(15)) and are simply shown schematically in Figure 6.

(8) $$\left(\mathrm{photocatalyst}\right)+\mathrm{hv}\to h_{\;\mathrm{VB}}^{+}+e_{\;\mathrm{CB}}^{-}$$

(9) $$H_{2}O\to H^{+}+{\mathrm{OH}}^{-}$$

(10) $$e_{\;\mathrm{CB}}^{-}+O_{2}\to \cdot O_{2}^{-}$$

(11) $$\cdot O_{2}^{-}+H^{+}\to {\mathrm{HO}}_{2}^{\cdot }$$

(12) $${\mathrm{HO}}_{2}^{\cdot }+{\mathrm{HO}}_{2}^{\cdot }\to H_{2}O_{2}+O_{2}$$

(13) $$e_{\;\mathrm{CB}}^{-}+H_{2}O_{2}\to \cdot \mathrm{OH}+{\mathrm{OH}}^{-}$$

(14) $$h_{\;\mathrm{VB}}^{+}+{\mathrm{OH}}^{-}\to \cdot \mathrm{OH}$$

(15) $$h^{+}/\cdot O_{2}^{-}/{\mathrm{HO}}_{2}^{\cdot }/\cdot \mathrm{OH}+\mathrm{pollutant}\to \mathrm{products}\left({\mathrm{CO}}_{2}+H_{2}O\right)$$

The mechanism of semiconductor photocatalysis is well understood. In summary, when the photocatalyst is irradiated under visible light, electrons (e^−^) are promoted from the valence band (VB) to the conduction band (CB) of the samples, leaving an electron vacancy or hole (h^+^) in the VB (Equation (8)) as the absorption of photon energy exceeds or is equal to the forbidden band gap energy of photocatalyst semiconductor materials [27]. As long as the separation of the photogenerated charge carriers (e^−^ and h^+^) is retained, e^−^ and h^+^ then transfer to the surface of the photocatalyst and are engaged in redox reactions (electrons in the conduction band can be rapidly trapped by molecular oxygen adsorbed on the photocatalyst particle, which is reduced to form superoxide radical anions ($\cdot O_{2}^{-}$) (Equation (10)) that may further react with H^+^ to produce hydroperoxyl radicals (${\mathrm{HO}}_{2}^{\cdot }$) (Equation (11)) and further electrochemical reduction yields H_2_O_2_ (Equation (12)), leading to the generation of active species such as superoxide radical anions ($\cdot O_{2}^{-}$), hydroxyl radicals (·OH), and other strongly oxidizing free radicals that participate in the oxidation of organic pollutants such as RhB and can be decomposed to intermediates or mineralized products [28].

It is worth mentioning that the recombination of photogenerated charge carriers is still the main limitation factor in semiconductor photocatalysis because it lowers the whole quantum efficiency [29]. Clearly, insignificant degradation is shown in Figure 4A when pure Bi_2_Te_3_ NRs were used as the photocatalyst, which indicated that the life of photogenerated charge carriers in the pure Bi_2_Te_3_ structure was very short. The speculative main reasons, in comparison to nanoribbon or other ultrathin structures, for one thing, is that the specific surface area of pure Bi_2_Te_3_ is smaller so that the adsorbed species such as molecular oxygen were quite few in number so could not effectively capture the carriers, and for another, the distance photogenerated charge carriers transform to the surface is farther, so these two aspects improved the recombination chance of the charge carriers. Therefore, the exited electrons were more prone to revert to the VB without reacting with the adsorbed species and dissipate the energy as light and heat [30,31]. Furthermore, a Bi_2_Te_3_/Bi core-shell nanostructure can be generated by controlling the ratio of Bi_2_S_3_-to-Te. Moreover, according to the mechanism speculated above, even though the Bi_2_Te_3_ core is covered by a layer of Bi shell, e^−^ exits from the VB to the CB of Bi_2_Te_3_ under visible light, and e^−^ can further rapidly migrate from the Bi_2_Te_3_ core to the metallic Bi shell support, as shown in Figure 6A. The metallic Bi on the surface provided electron transport channels for further photochemical reactions. Thus, the Bi shell covering the surface of Bi_2_Te_3_ can enhance the photocatalytic activity under visible light by acting as an electron trap, promoting interfacial charge transfer and spatial separation, therefore delaying recombination of the e^−^ −h^+^ pairs and making full use of the electrons in the whole process [32,33]. After that, heterojunction coupling in photocatalysts has also been proven to be one of the most promising ways to prepare advanced photocatalysts because of its feasibility and effectiveness for the spatial separation of e^−^–h^+^ pairs, reducing recombination and therefore improving the photocatalytic activity. Figure 6B reveals a typical heterojunction structure mechanism for photocatalysis. To investigate the photocatalytic mechanism of Bi_2_Te_3_/AgBiTe_2_ in depth, the conduction band and valence band-edge positions of the catalyst can be seen through the Mott–Schottky diagram calculated by roughly using the Mulliken electronegativity [34,35,36] and the result of the UV–Vis measurement as the following Equations (16)–(18):

(16) $$\chi_{a}=0.5\left(A_{f}\right)+I_{1}$$

(17) $$E_{CB}=\chi_{M}+E^{0}-0.5E_{g}$$

(18) $$E_{VB}=E_{CB}+E_{g}$$

where $\chi_{a}$ is the absolute electronegativity of atoms, which can be expressed as the arithmetic mean of the atomic electron affinity and first ionization potential; $\chi_{M}$ is the electronegativity of the material, which can be regarded as the geometric mean of the absolute electronegativity of the constituent atoms; $A_{f}\;$is the atomic electron affinity; $I_{1}\;$is the first ionization potential;$\;E_{g}$ is the band gap energy of the material; $E_{CB\;}$ is the conduction band energy; $E_{VB\;}$ is the valence band energy; and $E^{0}$ is −4.5 eV related to the normal hydrogen electrode. In combination with the dates provided in Figure 6C and making use of the equations above, the calculated value for $E_{CB\;}$ and $E_{VB\;}$ are shown in Figure 6C. The CB and VB levels of AgBiTe_2_ were higher than the corresponding levels of semiconductor Bi_2_Te_3_; thus, the photogenerated electrons and holes will migrate to the CB of Bi_2_Te_3_ and the VB of AgBiTe_2_ under light irradiation, respectively, resulting in spatial separation and accumulation of photogenerated charge carriers. Their negative CB potential moved to −0.025 eV from −0.065 eV, and the positive VB potential shifted to 0.85 eV from 0.765 eV, respectively, which means that the redox potential for photoinduced charge carriers were all enhanced. A series of photochemical reactions were enhanced due to the unique structure, and all of the e^−^, h^+^, or other oxidation radicals were also been fully utilized, which all improved the catalytic oxidation ability of heterojunction photocatalysts and the photodegradation of organic pollutants.

**Figure 7:**
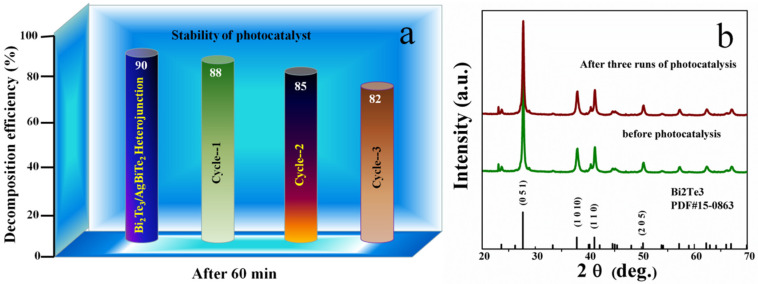
(**a**) Decomposition efficiency of Bi_2_Te_3_/AgBiTe_2_ heterojunction nanostructures with an increasing number of catalytic cycle, (**b**) XRD patterns of Bi_2_Te_3_/AgBiTe_2_ heterojunction nanostructures before and after photocatalytic runs.

In addition, for photocatalytic activity, the stability of catalysts is also one of the most concerning issues for their potential applications. It is vital to explore the photostability and recyclability of the Bi_2_Te_3_/AgBiTe_2_ heterojunction nanostructure photocatalyst, as it could appreciably reduce the dissipation over the whole photocatalytic process. Thus, we carried out three successive cycling runs of photodegradation of the RhB solution. As revealed in Figure 7, an imperceptible reduction (ca. 8%) was seen by analyzing the recyclability results of the Bi_2_Te_3_/AgBiTe_2_ heterojunction nanostructure for the photodegradation of RhB under visible light irradiation, which could prevent unnecessary loss in the recycling process. On the other hand, as illustrated in the XRD data (Figure 7b), there was no recognizable change in the pattern after three successive cycles of photocatalysis. Therefore, the cycling results reflect the stability of Bi_2_Te_3_/AgBiTe_2_ heterojunction nanostructure photocatalysts and hint at potential applications for environmental purification.

Herein, our point is the development of visible light functional heterojunction nanostructures that suppress the recombination of photoinduced charge carriers and offer a commendable photocatalytic performance for the decomposition of organic pollutants such as RhB. Furthermore, the results from the stability and recyclability experiments of the Bi_2_Te_3_/AgBiTe_2_ heterojunction nanostructure revealed their feasibility for potential environmental applications. In this paper, we expect that this research could obtain tremendous possibility in the photocatalysis field for the development of efficient, stable, and recyclable Bi_2_Te_3_/AgBiTe_2_ heterostructures.

## 4. Conclusions

In summary, nanocomposites with different structures including pure Bi_2_Te_3_, Bi_2_Te_3_/Bi core-shell, and Bi_2_Te_3_/AgBiTe_2_ heterojunction coupling nanorods were successfully prepared by hydrothermal synthesis using Bi_2_S_3_ as a template. This work provides a potential platform for the retardation of the recombination rate of photoinduced charge carriers in the Bi_2_Te_3_/AgBiTe_2_ heterostructure. At the same time, the prospect of photocatalysis in pure, core-shell, and heterojunction coupling nanostructures has been probed. Finally, it is also found that the Bi_2_Te_3_/AgBiTe_2_ heterostructure could offer superb photocatalytic performance over the corresponding pure or core-shell nanostructure for RhB degradation under visible light illumination. The suitable photocatalytic performance of the Bi_2_Te_3_/AgBiTe_2_ heterostructure could be hinged to its heterojunction structure geometry, which enhanced the interfacial contact, thus ensuring fast transportation and a lower recombination rate of photogenerated charge carriers. The above results illustrate new points in the photocatalysis field for exploring promising heterojunction nanostructures for environmental applications.
